# Authorship identification of documents with high content similarity

**DOI:** 10.1007/s11192-018-2661-6

**Published:** 2018-02-02

**Authors:** Andi Rexha, Mark Kröll, Hermann Ziak, Roman Kern

**Affiliations:** Know-Center GmbH, Inffeldgasse 13, Graz, Austria

**Keywords:** Writing style analysis, Content agnostic stylometry, High content similarity, Authorship identification

## Abstract

The goal of our work is inspired by the task of associating segments of text to their real authors. In this work, we focus on analyzing the way humans judge different writing styles. This analysis can help to better understand this process and to thus simulate/ mimic such behavior accordingly. Unlike the majority of the work done in this field (i.e. authorship attribution, plagiarism detection, etc.) which uses content features, we focus only on the stylometric, i.e. content-agnostic, characteristics of authors. Therefore, we conducted two pilot studies to determine, if humans can identify authorship among documents with high content similarity. The first was a quantitative experiment involving crowd-sourcing, while the second was a qualitative one executed by the authors of this paper. Both studies confirmed that this task is quite challenging. To gain a better understanding of how humans tackle such a problem, we conducted an exploratory data analysis on the results of the studies. In the first experiment, we compared the decisions against content features and stylometric features. While in the second, the evaluators described the process and the features on which their judgment was based. The findings of our detailed analysis could (1) help to improve algorithms such as automatic authorship attribution as well as plagiarism detection, (2) assist forensic experts or linguists to create profiles of writers, (3) support intelligence applications to analyze aggressive and threatening messages and (4) help editor conformity by adhering to, for instance, journal specific writing style.

## Introduction

Identifying and attributing authorship of different text passages can be beneficial for various tasks and areas including bibliometrics, information retrieval and plagiarism detection. In previous work, we illustrated how to incorporate ideas of authorship attribution into the mentioned areas. We introduced the concept of extending the retrieval process by including authorship information to allow for identifying text passages written by a particular author. In Rexha et al. (2015), we applied text segmentation to identify potential author changes within the main text of a scientific article. Rexha et al. (2016) presented a new feature representation of scientific documents that capture the distribution of stylometric features across the document and to predict the number of authors accordingly.

This follow-up paper [an extension of Rexha et al. (2017)] investigates the extent with which content-agnostic, stylometric features are capable of distinguishing between authors. We analyze the way human evaluators^1^ judge and assign similar writing styles with respect to text passages exhibiting a high content similarity. In contrast to related work, we thus focus on pure stylistic characteristics of authors, which usually tend to generalize better if the topic changes. Consider following two examples:

### *Example 1*

Stylometry is the application of the study of linguistic style, usually to written language.

### *Example 2*

Stylometry is the application of the study of linguistic style. It is usually applied to written language.

The information of these two sentences is the same, yet they slightly differ in the way, how this information is conveyed. In Example 1, the author appears to favor longer sentences; in Example 2 shorter sentences. In case of these constructed examples content features will not be sufficient to distinguish between authors; to do that we need to focus on content-agnostic, i.e. pure stylometric features. Consider the case of a journal hiring a new employee and asking her to adhere to a (journal-) specific style in her articles. Providing a tool that compares her writing style to the journal style can shorten the settling in process. The same logic applies to intelligence agencies to support the analysis of aggressive and threatening messages, i.e. identify the responsible author. Furthermore, commercial products (i.e. Grammarly)^2^ that help in writing can incorporate such mechanisms and thus assist authors to keep the same writing style throughout the document. Finally, these findings can also help to build systems supporting forensic experts with suggestions for candidates of plagiarism.

As a first step towards building such a system, we conducted two different pilot studies to understand whether humans are capable of distinguishing between writing styles without considering the content as a discriminating feature. As already mentioned, this makes our study different from most of plagiarism detection tasks. The first is a quantitative study with annotators from the crowdsourcing platform CrowdFlower. This study has the disadvantage of the unknown quality of the judgments. Even though we use mechanisms to avoid random choices from the crowd, we cannot fully prevent it. To overcome such a problem, we performed a qualitative study, which represents the main contribution of this article. In this study, the current authors evaluate the list of experiments and give a detailed description of the thinking process while annotating.

The outcome of the two studies was quite similar. Even though it appears more likely not to have random evaluations (72% confidence), our findings revealed that this task is challenging, even for humans. We also conducted an exploratory data analysis where we statistically compared the decisions against content features and content-agnostic features. We didn’t find any correlation that explains the different judgments between annotators. In addition, we make our dataset publicly available.^3^

## Related work

Over the past decades, we have observed an ever-growing amount of scientific output; much to the joy of research areas such as *bibliometrics* as well as *scientometrics* which both aim to measure and quantify the scientific output. This ongoing growth of the volume of scholarly publications poses significant challenges leading to the incorporation of ideas from other fields such as computer linguistics to improve and enhance the measuring and analysis processes. In recent work, we proposed to add the concept of author attribution into the pre-processing of the analysis of scientific publications. In Rexha et al. (2015), we focused on attributing particular segments of an article to individual authors. We thereby initiated a discussion on the implicit definition of scientific authorship; to give an example, in many scientific domains it is assumed that the first author did most of the (writing) work and the last author contributed ideas being the head of the research group. Follow-up work (cf. Rexha et al. 2016) studied the distribution of stylometric features across a scientific article to predict the number of authors accordingly. The classification performance then represents so-to-say a quantification of the amount of information that is contained within the stylometry of a scientific article about the number of authors involved in writing it.

Authorship attribution (cf. Stamatatos 2009; Juola 2008) can be expressed as classification task where, from a set of candidate authors the author of a disputed article is to be identified. This line of research can be traced back to the 19th century when Mendenhall (1887) aimed to characterize the plays of Shakespeare. A century later Mosteller and Wallace (1964) used a Bayesian approach to analyze ’The Federalist Papers’—one of the first authorship disputes in literature. Since then, a line of research known as stylometry focused on defining features to quantify an author’s writing style (cf. Holmes 1998) including (1) lexical features such as average word/sentence length and vocabulary richness, (2) syntactical features such as frequency of function words and use of punctuation, and (3) structural features such as indentation. In terms of supervised classification, it translates into the task of proper feature selection/extraction (cf. Stamatatos 2009). This process of selecting features is often closely related to the research or application scenario at hand, i.e. adapted to domains, genres or textual characteristics. Attributing authors being faced with short messages (cf. Villar-Rodriguez et al. 2016; Brocardo et al. 2013) differs from being faced with unstructured texts (cf. Zhang et al. 2014).

With the advent of social media, in particular, the way our society communicates and exchanges information has changed. Social media opens up new opportunities to express opinion. The process of determing authors of online messages, especially those with offensive as well as threatening expressions, is thus given higher priority. Sousa Silva et al. (2011) propose a set of stylistic markers for automatically attributing authorship to micro blogging messages such as Twitter. In their classification setting, they are investigating whether ’non-traditional’, content-agnostic markers such as emoticons contain relevant information for the task. Inches et al. (2013) and van der Knaap and Grootjen (2007) conduct authorship attribution analyses in chat logs exploring statistical approaches as well as formal concept analysis respectively. With respect to cybercrime, Iqbal et al. (2013) experiment with different settings including the characterization of authorship. They present a data mining approach that uses frequent stylometric patterns, i.e. a combination of stylometric feature items that occurs frequently.

Adding information on authorship can also be considered as adding semantic information, thus supporting the analysis of scientific publications. To be of value, scientific publications are subjected to semantic enrichment in various ways. Adding semantics includes, for instance, assigning instances to concepts which are organized and structured in dedicated ontologies. Entity and relation recognition thus represent a vital pre-processing step. To give an example, medical entity recognition (cf. Abacha et al. 2011) seeks to extract instances from classes such as “Disease”, “Symptom” or “Drug” to enrich the retrieval process. Research assistants such as BioRAT (cf. Corney et al. 2004) or FACTA (cf. Tsuruoka et al. 2008) then can offer an added value employing this type of semantic information. Liakata et al. (2012) departed from a mere content-level enrichment and focused on the discourse structure to characterize the knowledge conveyed within the text. For this purpose, they identified 11 core scientific concepts including “Motivation”, “Result” or “Conclusion”. In the Partridge system, Ravenscroft et al. (2013) built upon the automated recognition to automatically categorize articles according to their types such as Review or Case Study. The TeamBeam (cf. Kern et al. 2012) algorithm aims to extract an article’s meta-data, such as the title, journal name and abstract, as well as explicit information about the article’s authors. Implicit information about an author includes her writing style, which reflects among others, the writer’s personality as well as directly relates to characteristics such as readability and clarity. Stylometry represents the line of research which focuses on defining features to quantify an author’s writing style (cf. Holmes 1998). Bergsma et al. (2012) used stylometric features to detect the gender of an author and to distinguish between native versus non-native speakers and conference versus workshop papers.

## Experimental setup

**Fig. 1:**
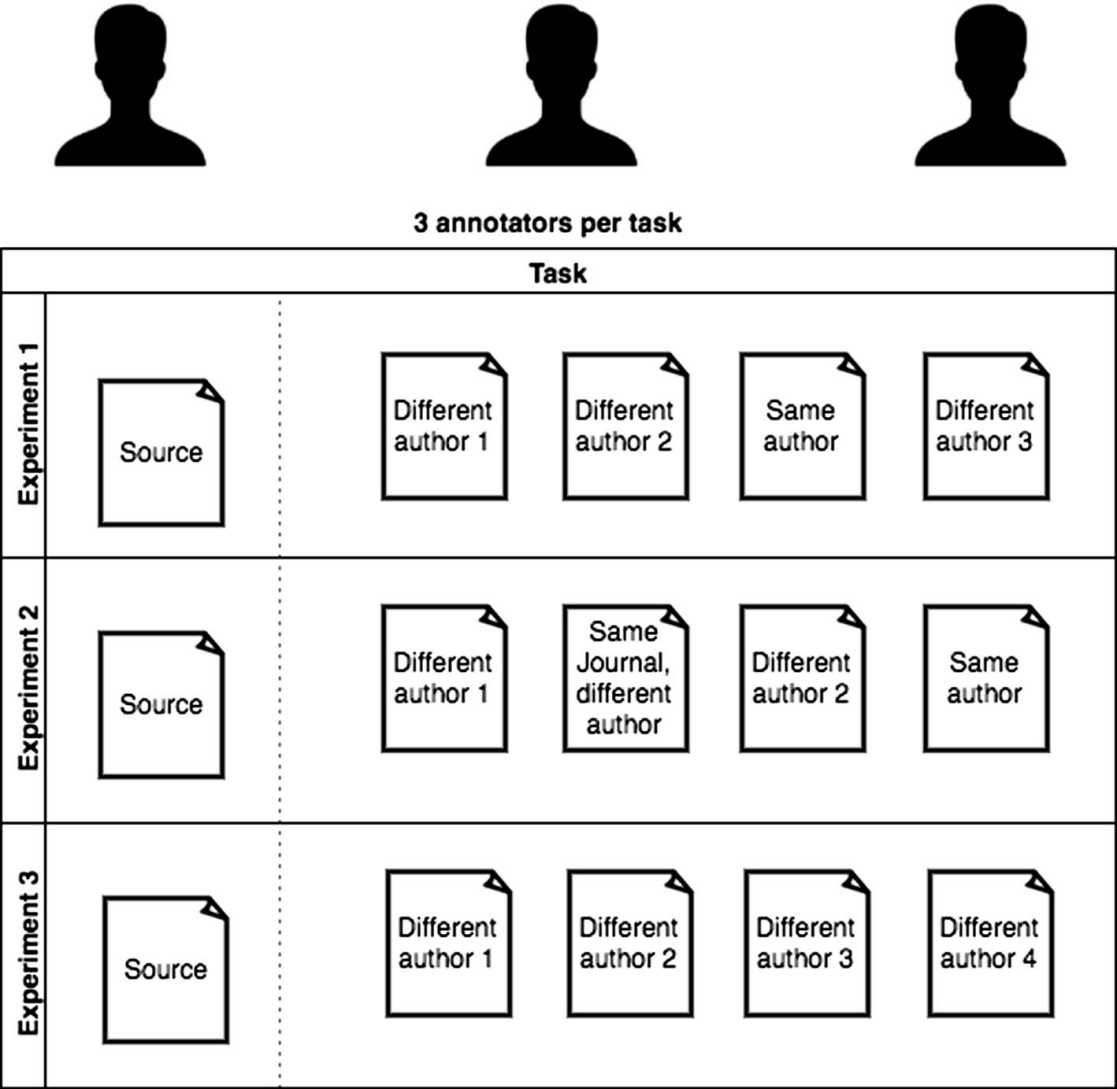
Description of a task. Three evaluators are assigned to each task and rank each target of the experiment from the “most similar” to the “least similar”

In order to understand whether humans can identify the authorship once the content information has been removed, we conducted two pilot studies. In these studies, we provided human annotators with one source and four target textual snippets in different experiments. In the first experiment, one of the targets is written by the same author as the source, and the other three are written by different authors as the source. Then, we have the annotators rank the snippets from the most to the least similar concerning the writing style, asking them to classify the target written by the same author as “most similar” (see Fig. 1). Since we wanted to extract all the clues about the stylometry, we forced users to rank the articles avoiding to provide them with options like “not able to find”.

For the studies, we selected data from Pubmed,^4^ a free database created by the US National Library of Medicine. This database holds full-text articles from the biomedical domain together with a standard XML markup that rigorously annotates the complete content of the published document. It also contains valuable metadata about the authors and the journal in which the article is published. At first, we retrieve documents written by only a single author to obtain pure writing styles. Note that some articles could be written by ghostwriters or by colleagues of authors helping them with English writing. In some other cases, the institution provides the authors with editing services, blurring the real style of the authors. Another drawback of this hypothesis relies on the possible change of the writing style in distance of years. We intend to address such shortcomings in our future work.

From the previously selected documents, we chosen a subset and decided to make the annotators rank text snippets which are drawn from the beginning of the introduction section (we select the first sentences until the one ending after the 400-th character). The rationale behind this choice is twofold: (1) it gets more and more difficult for the user to remain focused on the task while reading a long text; (2) we hypothesize that the introduction contains less topological information than other parts of the scientific papers. Having selected the text snippets, we designed three experiments (which we call a task) for each annotator. For each of the experiments we presented the annotators with a source and four target snippets, subject to different problem settings (see Fig. 1):

- In Experiment 1, we presented a target snippet written by the same author as the source as well as three others written by different authors as the source.
- In Experiment 2, we provided the annotators with one target article written by the same author as the source, one target article from a different author but published in the same journal as the source and two target articles written by different authors and published in different journals as the source. This experiment is designed to capture any correlation between the writing style within the same journal, presumably within the same scientific topic.
- In Experiment 3, we wanted to gain as much information as possible from the users’ thinking while ranking. Thus, we showed four target snippets written by different authors as the one of the source snippet, while still suggesting to the annotator that one of the targets is written by the same author as the source.

In the last design step of our pilot study, we selected 90 random snippets from the PubMed database as candidate source snippets. We indexed the database of the snippets from single authors by stemming the words and removing the stop-words. We assigned 30 snippets to each of the categories of the experiments, and we performed for each of them a search according to the request:

- In Experiment 1, we searched for 10 similar articles from the same author and 100 from different authors.
- In Experiment 2, we searched for 10 similar articles from the same author, 10 from the same journal but different author, and 100 from different authors and journals.
- In Experiment 3, we searched for 100 similar articles from different authors.

Based on these results, we performed a cosine similarity between the vector of the words with the source snippet and selected the most similar ones according to the experiment description. This way, the content information should have been removed as a source of information for authorship identification. For example, for Experiment 1 we selected the most similar article from the same author and three from different authors. Additionally, we also performed a manual check and removed the snippets that we assumed contained content hints for texts written by the same author (mainly based on keywords or phrases). At the end of this phase, we chose 66 experiments (22 per each category of experiments previously described).

Once we build the experiments, we made two different studies. The first was quantitative (due to the vast amount of users), performed using the crowd-sourcing platform CrowdFlower.^5^ The platform provides workforce from different countries helping to label and to enrich data. Here, we presented the same set of experiments (task) to three different annotators (see Fig. 1). Although we have used some mechanisms to avoid random selection, it is almost impossible to assure it.

In the second study, we perform a qualitative experiment, where the authors of this paper evaluated all the experiments. We call it qualitative due to the quality assurance from the annotators. Three of the users, were asked to perform a stylometric ranking, and the forth to focus on the content. Furthermore, we split the set of the experiments in two equal parts. In the first part, we presented to the user the same experiments as shown to the crowd. In the second one, we added a list of features that might help the evaluators with the ranking. We extracted the following features for each of the text snippets:

- Numeric Features, which contain numeric information about the writing style. We used the features listed in the Table 2. We augmented this list with the “depth of the dependency trees”(indicating the maximum depth of the dependency tree in each sentence) as well as with “stop words ratio” (indicating the ratio of the stop words with the total number of tokens in each sentence).
- Common selected phrases, representing a list of selected phrases present in both, source and target snippet, like “although”, “most common”, etc.
- Descriptive features, informing whether the first sentence of the source and the target starts with a definition (for example: “Breast cancer is...”) or whether hyphens are used in both snippets.
- Outliers, representing a list of Numeric Features that have a larger value than the 95th percentile and smaller than the 5th percentile of the distribution in all the snippets of the experiments.

**Fig. 2:**
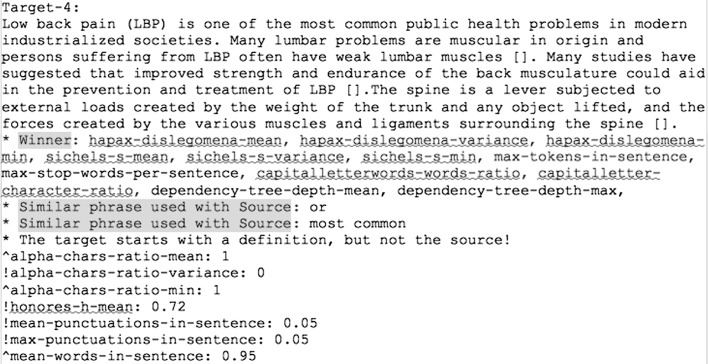
Example of a target snippet presented to the users in the qualitative evaluation. The list of features is added to half of the experiments to help the ranking process

Then, we presented them to the users (see Fig. 2). For the numeric features, for each target, we showed to the user the closest value with the source. We indicate them as “Winners”. We also present the list of “Common selected” phrases and “Descriptive features”. Lastly, we showed the Outliers denoting with “!” the features with smaller values than the 5th percentile and with “$\wedge$” for values with larger values than the 95th percentile of the distribution.

## Results

### Pilot study #1

The job in CrowdFlower was performed by 56 different annotators from 29 different countries. Since our goal is to rank based on the writing style, the level of understanding English isn’t of a big concern for the task. To avoid random selection, we configure the system to disallow annotation in less than 20 s. At first glance, the annotators have a small agreement in the ranking of the similarity between source and target snippets. Without considering the rank itself, a full agreement is achieved in 26 targets, 160 have an agreement of two annotators, and 78 of the targets have no agreement at all. For a more detailed analysis, we use Krippendorff’s alpha (cf. Krippendorff 2004) measure to determine the inter-rater agreement for the ranking of each target. This was computed using the library “DK-Pro statistics”.^6^ The results show:

- An inter-rater agreement of 0.299
- An observed disagreement of 0.699
- An expected disagreement of 0.999

We continue to explore the annotator’s rank by considering the snippets written by the same author and those written within the same journal (but by different authors). Table 1 shows the amount of times users selected the articles in each category of similarity. With a random selection, the expected precision is of 25%.

**Table 1:** Pilot study #1: ranking of the crowd-sourcing’ evaluators for snippets from the same author and snippets from the same journal but different author

Snippet/ranking	Most similar (%)	Similar (%)	Less similar (%)	Least similar (%)
Same author	19	45	24	12
Same journal	21	41	26	12

The expectation for a random selection is 25%

First, we select a list of stylometric features to extract from the source and the target texts. The literature suggests a broad amount of stylometric features (cf. Mosteller and Wallace 1964; Tweedie and Baayen 1998 or Stamatatos 2009). We filter the content features and some of those who do not make sense in short texts. Table 2 presents the list of features we extract for each snippet. In addition, we calculate the minimum, maximum, average and variance for each of those features across every snippet.

**Table 2:** List of stylometric features used calculate the similarity between text snippets

Feature name	Description
Alpha-chars-ratio	The fraction of total characters in the paragraph which are letters
Digit-chars-ratio	The fraction of total characters in the paragraph which are digits
Upper-chars-ratio	The fraction of total characters in the paragraph which are upper-case
White-chars-ratio	The fraction of total characters in the paragraph which are whitespace characters
Type-token-ratio	Ratio between the size of the vocabulary (i.e. the number of different words) and the total number of words
Hapax-legomena	The number of words occurring once
Hapax-dislegomena	The number of words occurring twice
Yules-k	A vocabulary richness measure defined by Yule
Simpsons-d	A vocabulary richness measure defined by Simpson
Brunets-w	A vocabulary richness measure defined by Brunet
Sichels-s	A vocabulary richness measure defined by Sichel
Honores-h	A vocabulary richness measure defined by Honore
Average-word-length	Average length of words in characters
Average-sentence-char-length	Average length of sentences in characters
Average-sentence-word-length	Average length of sentences in words

Many of those features are defined in Tweedie and Baayen (1998)

We consider the similarity between the source and the targets as a cosine similarity between the feature vectors. Formally, if $V_1=[v_1,\ldots , v_n]$ is a vector of the features $v_1, \ldots , v_n$ and $V_2=[{v_1}_2, \ldots ,{v_n}_2]$ is a vector of the features ${v_1}_2, \ldots , {v_n}_2$, their cosine similarity is defined as:

$$\begin{aligned} {\hbox {similarity}} = \cos \left( V_1, V_2\right) =\frac{\sum _{i=1}^{n}v_i \cdot {v_i}_2}{\sum _{i=1}^{n}v_i \cdot {\sum _{i=1}^{n}{v_i}_2}} \end{aligned}$$

**Fig. 3:**
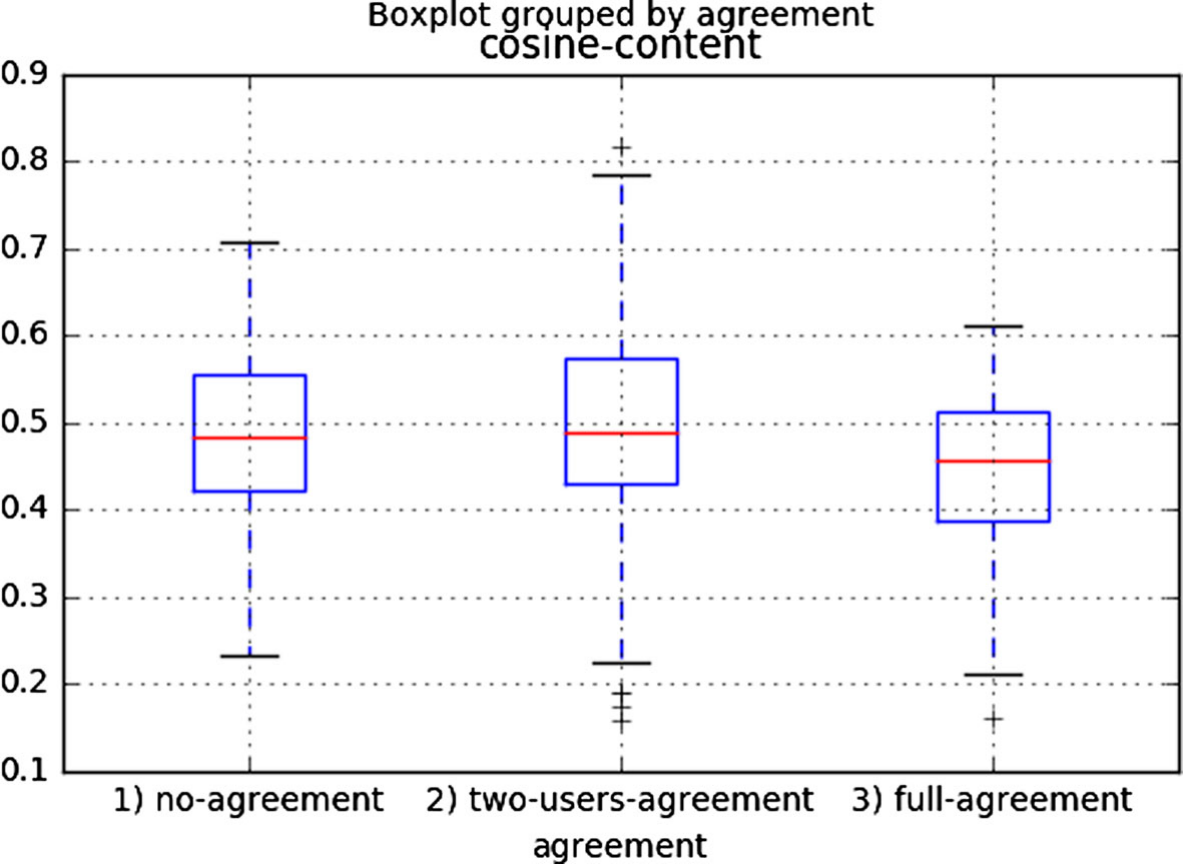
Box plots representing the distribution of the annotators’ agreement over the similarity of the content

**Fig. 4:**
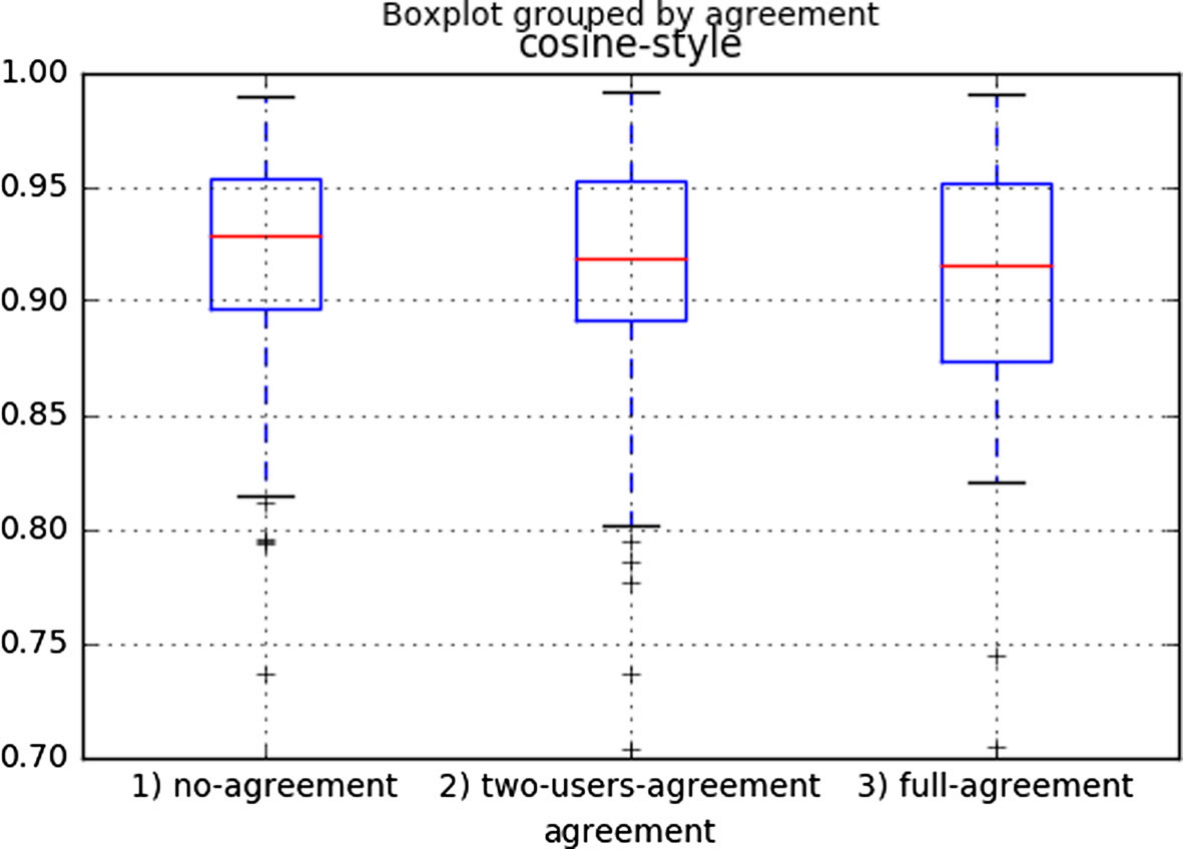
Box plots representing the distribution of the annotators’ agreement over the similarity of the writing style

As depicted in Fig. 3, we created box-plots to study whether there is a correlation between the user agreement and the content similarity and in Fig. 4 one between the user agreement and the writing style similarity.

**Fig. 5:**
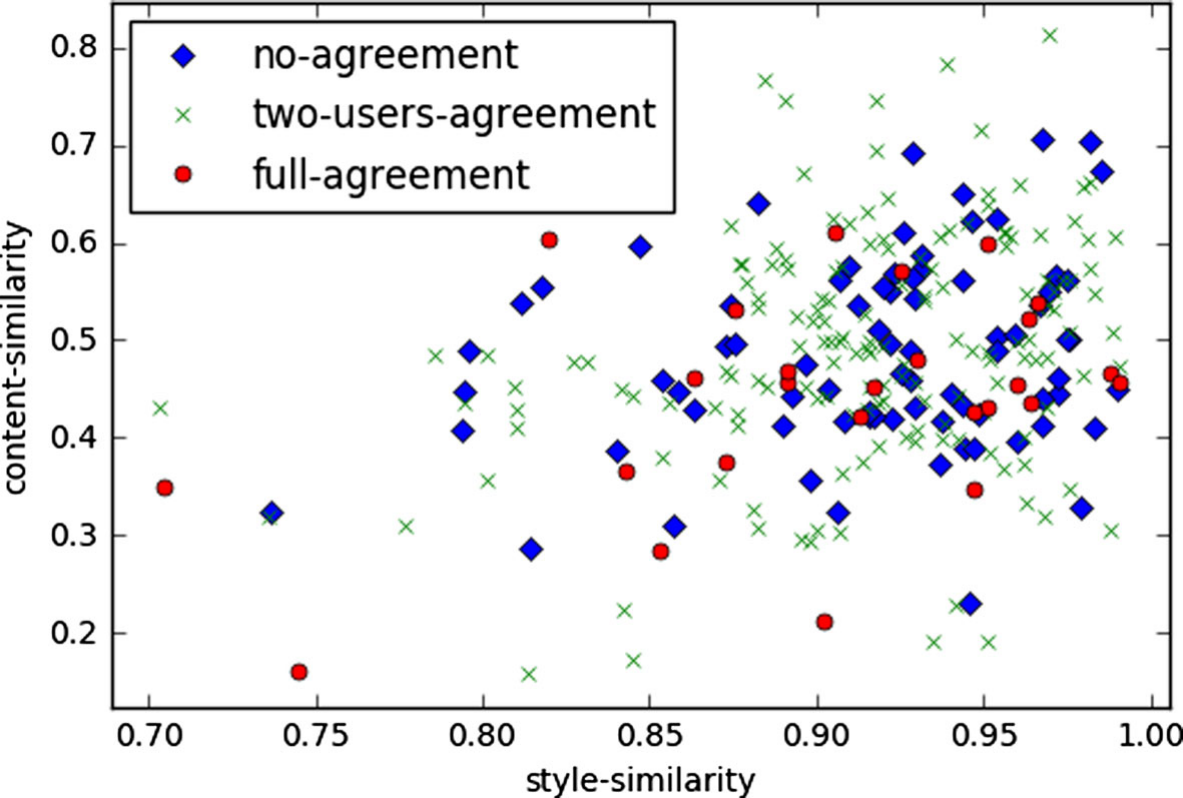
Scatter plot relating three dimensions: style similarity, content similarity and agreement between annotators

There is no clear evidence that explains the agreement/disagreement among annotators from the considered features. To dig more deeply, in Fig. 5 we created a scatter plot to comprehend whether there is a correlation between the three similarities, i.e. content similarity, the writing style similarity and the inter-rater agreement.

The scatter plot does not provide any visual hint about the annotators’ agreement/disagreement. In addition, we plotted every combination considering, instead of the whole vector of the aforementioned features (see Table 2), each of them singularly. Yet, we did not notice any clear pattern. As there is no additional information added to the previous plot we omitted them in this paper.

Finally, we empirically measured whether the annotators did their ranking in a random manner. We executed 500.000 rounds of random studies and, for each of them, calculated the inter-rater agreement using the Krippensdorff’s alpha. The results show an average of 0.250 with a variance of 0.020. 28% of the cases have a larger agreement than our study, thus we can conclude with a confidence of 72% that the annotators in our experiment did not rank in a random manner.

### Pilot study #2

In this study, the evaluators are the authors of the current paper. Here, we try to avoid random selection which may be present in the crowd-sourcing platforms. As already mentioned, we showed two different types of experiments to the users. The first type was the same as the one presented to the crowd. In the second, we enrich the presented snippet with a list of features that might help the annotator decide. The first three annotators used the writing style as a feature for ranking the snippets, while the last one used the content information. Table 3 shows for each annotator, the precision in finding the exact author. The precision for a random selection has an expectation of 25%.

**Table 3:** Pilot study #2: precision of the annotators in finding the same author as the source snippet

	Precision without features	Precision with features
Annotator #1	0.22	0.22
Annotator #2	0.27	0.29
Annotator #3	0.16	0.25
Annotator #4	0.11	0.15

Results are presented for experiments provided with and without features to help for the ranking (the expectation of the precision for a random selection is 25%). Please note: Annotator #4 performed the ranking task wrt. content features

As we can see, the results show a slight improvement in the case the evaluators use the extracted features, but it isn’t clear if this effect is a result of the additional information presented. To ensure that the content was not key to identifying the correct author, Annotator #4 intentionally conducted the experiments based on the content resulting in the worst precision values. We collected the observation from each of the annotators and present them below. These can serve either as confirmation of existing features, or as inspirations for new features for automatic authorship identification tasks.

#### Annotator #1

The first observation, which is specific to the data set is the initial sentence of the paragraph. Often authors tend to give a definition of the key terms of the text, for example the sentence may start with “Breast cancer is the most common cancer [...]”. Other authors tend to use more active voice to raise the awareness via phrases like “Break cancer remains the most common cancer”. The last example also represents a writing style which makes use of temporal aspects, which includes phrases like “since”, “more recently”, “recent advances in [...]”, or specific dates like “in 2013”.

Another observation that might be specific to the data set is the varying degree of granularity for named entities, for example “Liver cancer”, in contrast to “Primary liver cancer”. Related to this is the spelling of certain entities, for example one author may write “Castelman’s disease”, while others may refer to the same concept as “Castelman disease”. The same also applies to the capitalization of concept, for example “short linear motifs”, or “Short Linear Motifs”. To a lesser extend, this has also been observed for abbreviations (“HIV1”, “hiv”, “HIV-1”).

Specifically in scientific literature, there are only a few authors that make use of words that are considered too emotional or imprecise, for example “tremendous” or “dramatic”. The level of preciseness also varies between authors, some may state “approximately 500,000 cases”, while others try to be more exact, for example “528,000 cases”. Another potential indicator for specific authors is the use of “slang abbreviations”, for example “can’t” instead of “cannot”. Given that authors are consistent and use the same spelling for all their written text, different authors could be distinguished. This also applies to synonyms (“high blood pressure” vs. “hypertension”) or alternative spellings (“etiology” vs. “aetiology”).

The initial words of a sentence often appear to convey information about the specific writing style. Some authors use phrases to link two sentences. Examples are: “Indeed”, “Although”, “Hence”, “Thus”, “While”, “Moreover” and many more. The usage of such words or just specific words might be indicative of certain authors. Furthermore, the presence (or absence) of a comma following such words, might be a useful feature to distinguish two writing styles. Some authors also appear to have the initial words to indicate a temporal aspect, for example “Since then”.

Trigger phrases may also occur within the sentence, which are characteristic for specific writing style, for example: “not very long ago”. A special case of such phrase is “comprise”, where some authors add the word “of”. This might also be indicative for the distinction between native and non-native speakers. Native speakers may also tend to use words, which are less frequent. Another obvious difference is whether the author uses British or American spelling, which might also be due to the intended target audience. The way numbers are written might also be specific to the locale of the text in addition to the preference of the author, for example “55,000”, “55,000” or “55,000”. Similar to this feature is the usage of the “Oxford comma”, where a comma is also used for the last item in an enumeration (“foo, bar, and baz” vs. “foo, bar and baz”); its presence might help to separate two authors. In general, the usage of semicolons, dashes and brackets might also be specific to some authors.

Another feature, which requires a certain level of consistency is the usage of multi-word terms, which might be written as single word, separated by a hyphen or written as separate words. Examples for this observation are: “US-born” versus “US born”, “over weight” versus “overweight” and “crossing-over” versus “crossing over”.

#### Annotator #2

After getting an overview of the first example snippets, it appeared that the informative value of typical features like the comma usage, sentence length or usage of brackets was not sufficient to arrive at a conclusion. In particular when the style is, in some cases, dependent upon the publisher, which is typically the case in scientific literature (e.g. citation style). In general, it seemed that stylistic choices and preferences of the author had a bigger impact. And sometimes the distinct absence of particular styles was more informative. Some authors appear to favor including precise figures within their text while others tend to use descriptive language. (e.g. “about one-third”, “30%”, “30.2%”) In general, the usage, or their absence, of precise figures within the text seems to be a highly informative characteristic of the authors. Furthermore, some authors seem to have the tendency to list particular information, separated by commas, within their text, while others do not use this style at all. Another distinctive feature was the usage of abbreviations. First, some authors used a lot of abbreviations while others did not use them at all. Second, some defined these abbreviations and did not reuse them later in the text, although it would have been appropriate.

#### Annotator #3

The identification of the similarity in the writing style starts by analyzing the structure of the sentences for each snippet. The formulation of the first sentence is the main observed characteristic judged in a snippet. Authors expressing the same concepts once as a subject and once as an object leads to considering the texts as written by different authors. For example, “Both obesity and metabolic syndrome (MetS) are well known.” and “A major obstacle in the treatment of overweight and obesity is hunger.” express “obesity” as either the subject or the object.

In the cases where the first sentence starts similarly in various target snippets, the way it is expressed also provide a differentiating feature. Some authors favor details compared to the short sentence. Furthermore, the distinction extends to the structure of the snippet. Some authors tend to favor long sentences to short ones. The structure of these sentences is seen as a characteristic that helps to decide the similarity between the presented texts.

Other aspects that were used to judge were:

- The way numeric values are represented (some use a comma or dots: 400.000 vs. 400,000).
- Whether there was a different representation of statistics (some favor percentage to total amounts: example: 350.000 vs. 10% of female)
- The use of enumeration (some authors like to enumerate information, and some prefer to have a description for each of the details).

Also, the specific phrases used, gave hints about the authorship attribution. For example, phrases like, “which” or “although” were considered very informative.

#### Annotator #4

For the sake of comparison between rankings based on writing style versus content, we add the ranking approach of Annotator #4:

After the first ranking task, it became apparent that an understanding of the source’s content was essential to conduct the ranking. Similar to a summarization task, factual information was identified. This identification procedure can be best described as identifying answers to the 5W question types, i.e. who, what, when, where and how. It could be observed that the temporal dimension, for instance, the year the source’s facts were referring to, was helpful when ranking the targets. Comparing the facts from the source with the ones to be ranked turned out as the common procedure for the ranking; for this part the support of domain experts familiar with synonymous medical expressions would have sometimes been beneficial.

As a first step, targets which were off-topic or out-of-domain were placed at the bottom of the ranking. Then, a closer look was taken at the targets sharing most of the facts with the source. These targets were ranked according to the coverage of content, i.e. the number of shared facts. In some experiments for example, the targets were missing facts about a certain region a disease was found. In several cases, two targets shared an equal number of facts with the source; yet one of them offered more information, i.e. more facts than the other one. These cases were considered as less similar to the source as well. In cases where the amount of facts were more or less equal, the following two indicators influenced the ranking. First, the order in which the information is presented—same order as in the source equalled a higher similarity than a different order. Second, if the order of information was the same, readability tipped the scales in favor of the more readable than the less readable one.

## Conclusion and future work

In this paper, we have conducted two extensive studies to gain a better understanding (1) how humans judge an author’s writing style (being faced with text passages exhibiting a high-content similarity) and (2) which content-agnostic, stylometric features they preferably use to identify an author. These experiences and observations contribute to automate (mimick) this process by identifying as well as by distinguishing specific features used by humans in their decision making process. We provide detailed descriptions and observations of this author identification process which will prove valuable in an effort to develop algorithms, for instance, in areas like plagiarism detection or forensic analyses. Furthermore, our findings indicate that the task turns out to be very challenging, especially with the current experiment settings. The results also indicate that the annotation process from the crowd is more likely not to have random evaluations (72% confidence). In addition, we have made our data set publicly available to the research community to enable further investigations and algorithm development.

In future work, we plan to take the author’s institution into account to serve as a dimension in the paper selection process—similarly with papers published in the same journal. We also plan to take into account text passages written within a distance of years by the same author. We plan to extend our study by increasing and diversifying the set of experiments aiming to capture, from human annotators, properties of the thinking process while performing this task. We also intend to automatically learn and suggest personalized features for each of the annotators, helping them rank, according to their metrics.

## Footnotes

1 In this paper, we use “user”, “evaluator” and “annotator” as synonyms.

2 https://www.grammarly.com/.

3 https://zenodo.org/record/437461.

4 https://www.ncbi.nlm.nih.gov/pubmed.

5 https://www.crowdflower.com/.

6 https://dkpro.github.io/dkpro-statistics/.

## References

[CR1] Abacha, A., & Zweigenbaum, P., (2011). *Medical entity recognition: A comparison of semantic and statistical methods*. BioNLP 2011 Workshop. Association for Computational Linguistics.

[CR2] Bergsma, S., Post, M., & Yarowsky, D. (2012). Stylometric analysis of scientific articles. In *Proceedings of the conference of the North American chapter of the ACL: Human language technologies*.

[CR3] Brocardo, M., Traoré, I., Saad, S., & Woungang, I. (2013). Authorship verification for short messages using stylometry. In *International conference on computer, information and telecommunication systems (CITS)*.

[CR4] Corney, D., Buxton, B., Langdon, W., & Jones, D. (2004). BioRAT: Extracting biological information from full-length papers. In *AI-2013: The thirty-third SGAI international conference*. 10.1093/bioinformatics/bth386 15231534

[CR5] Holmes D (1998). The evolution of stylometry in humanities scholarship. Literary and Linguistic Computing.

[CR6] Inches, G., Harvey, M., & Crestani, F. (2013). Finding participants in a chat: Authorship attribution for conversational documents. In *International conference on social computing*.

[CR7] Iqbal F, Binsalleeh H, Fung B, Debbabi M (2013). A unified data mining solution for authorship analysis in anonymous textual communications. Information Sciences.

[CR8] Juola, P. (2008). *Authorship attribution*. Foundations and Trends R in Information Retrieval, 1.

[CR9] Kern R, Jack K, Hristakeva M, Granitzer M (2012). TeamBeam meta-data extraction from scientific literature. D-Lib Magazine.

[CR10] Krippendorff K (2004). Content analysis: An introduction to its methodology.

[CR11] Liakata M, Saha S, Dobnik S, Batchelor C, Rebholz-Schuhmann D (2012). Automatic recognition of conceptualization zones in scientific articles and two life science applications. Bioinformatics.

[CR12] Mendenhall T (1887). The characteristic curves of composition. Science.

[CR13] Mosteller F, Wallace D (1964). Inference and disputed authorship: The federalist.

[CR14] Ravenscroft, J., Liakata, M. & Clare, A. (2013). Partridge: An effective system for the automatic classification of the types of academic papers. In *AI-2013: The 33rd SGAI international conference*.

[CR15] Rexha, A., Klampfl, S., Kröll, M., & Kern, R. (2015). Towards authorship attribution for bibliometrics using stylometric features. In *Proceedings of the 1st workshop on mining scientific papers (ISSI)*.

[CR16] Rexha, A., Klampfl, S., Kröll, M. & Kern, R. (2016). Towards a more fine grained analysis of scientific authorship: Predicting the number of authors using stylometric features. In *Proceedings of the 3rd workshop on bibliometric-enhanced information retrieval (BIR)*.

[CR17] Rexha, A., Kröll, M., Ziak, H., & Kern, R. (2017). Extending scientific literature search by including the author’s writing style. In *Proceedings of the 5th workshop on bibliometric-enhanced information retrieval (BIR)*.

[CR18] Sousa Silva, R., Laboreiro, G., Sarmento, L., Grant, T., Oliveira, E., & Belinda, M. (2011). ‘twazn me!!!; (’Automatic authorship analysis of micro-blogging messages). In *16th international conference on applications of natural language to information systems*. NLDB.

[CR19] Stamatatos E (2009). A survey of modern authorship attribution methods. Journal of the American Society for Information Science and Technology.

[CR20] Tsuruoka Y, Tsujii J, Ananiadou S (2008). FACTA: A text search engine for finding associated biomedical concepts. Bioinformatics.

[CR21] Tweedie F, Baayen H (1998). How variable may a constant be? Measures of lexical richness in perspective. Computers and the Humanities.

[CR22] van der Knaap, L., & Grootjen, F. (2007). Author identification in chatlogs using formal concept analysis. In *Proceedings of the 19th Belgium–Netherlands artificial intelligence conference*.

[CR23] Villar-Rodriguez E, Del Ser J, Bilbao M, Salcedo-Sanz S (2016). A feature selection method for author identification in interactive communications based on supervised learning and language typicality. Engineering Applications of Artificial Intelligence.

[CR24] Zhang C, Wu X, Niu Z, Ding W (2014). Authorship identification from unstructured texts. Knowledge-Based Systems.

